# The unreachable doorbells of South Texas: community engagement in *colonias* on the US-Mexico border for mosquito control

**DOI:** 10.1186/s12889-022-13426-z

**Published:** 2022-06-13

**Authors:** Jose G. Juarez, Ester Carbajal, Katherine L. Dickinson, Selene Garcia-Luna, Nga Vuong, John-Paul Mutebi, Ryan R. Hemme, Ismael Badillo-Vargas, Gabriel L. Hamer

**Affiliations:** 1 Department of Entomology, Texas A&M University, College Station, TX USA; 2 Colorado School of Public Health, Aurora, CO USA; 3 Division of Vector Borne Diseases, Centers for Disease Control and Prevention, Fort Collins, CO USA; 4 Division of Vector-Borne Diseases, Centers for Disease Control and Prevention, Dengue Branch, San Juan, PR USA

**Keywords:** Community engagement, Autocidal gravid ovitrap, Autodissemination station, Mosquito, Vector control

## Abstract

Mosquitoes and the diseases they transmit continue to place millions of people at risk of infection around the world. Novel methods of vector control are being developed to provide public health officials with the necessary tools to prevent disease transmission and reduce local mosquito populations. However, these methods will require public acceptance for a sustainable approach and evaluations at local settings. We present our efforts in community engagement carried out in *colonias* of the Lower Rio Grande Valley in south Texas for mosquito surveillance, control, and ecological projects. Along the US-Mexico border the term *colonia* refers to impoverished communities that are usually inhabited by families of Hispanic heritage. The different engagements were carried out from September 2016 to February 2019; during this time, we had three distinct phases for community engagement. In Phase 1 we show the initial approach to the *colonias* in which we assessed security and willingness to participate; in Phase 2 we carried out the first recruitment procedure involving community meetings and house-to-house recruitment; and in Phase 3 we conducted a modified recruitment procedure based on community members’ input. Our findings show that incorporating community members in the development of communication materials and following their suggestions for engagement allowed us to generate culturally sensitive recruitment materials and to better understand the social relationships and power dynamics within these communities. We were able to effectively reach a larger portion of the community and decrease the dropout rate of participants. Progress gained with building trust in the communities allowed us to convey participant risks and benefits of collaborating with our research projects. Community engagement should be viewed as a key component of any local vector control program as well as for any scientific research project related to vector control. Even in the face of budgetary constraints, small efforts in community engagement go a long way.

**Supplementary Information:**

The online version contains supplementary material available at 10.1186/s12889-022-13426-z.

## Background

Mosquitoes are vectors of human parasitic and viral diseases that affect millions of people per year around the world [1]. They cause the highest burden of disease transmission to humans by an arthropod vector [2] and are a major public health threat [3]. In the case of container *Aedes* mosquitoes and associated *Aedes*-borne viruses like dengue and Zika, traditional vector control programs have fallen short [4, 5], partially because of population growth in urban areas, connectivity between communities [6], climate change [7], the ability of *Ae. aegypti* to adapt to urban environments [8] and insecticide resistance [9]. Surveillance of *Aedes* mosquitoes is a key component of any vector control program, but success varies depending on the type of surveillance and control tools used [10].

From the early 1950s to late 1980s, centralized control activities were very successful at reducing *Aedes aegypti* population in the Americas [11] and malaria transmission in Africa [12], as well as almost eliminating onchocerciasis transmission in West Africa [13]. However, the impact of these programs waned over time due to insecticide resistance, difficulty accessing houses (i.e., unwillingness to allow unknown technicians into homes and the smell of insecticides [14]), and lack of sustained investments [15]. Since the late 1990s, vector control programs in the Americas have become more decentralized, focusing on smaller areas, and using a bottom-up approach. More recently, programs have begun to emphasize engagement with community members and stakeholders as part of control efforts to improve long term sustainability of a project and help during control activities [16–19].

In the contiguous United States of America (USA), there are very few regions that have both presence of *Ae. aegypti* and local transmission of *Aedes*-borne viruses such as dengue or Zika. One of these regions is the Lower Rio Grande Valley (LRGV) located in south Texas [20]. Within the LRGV, mosquito control programs follow a decentralized regimen where the local cities or counties are responsible for surveillance and control [21]. In this region, vector control activities are minimally funded with an estimated $0.05 per person per year contributing to the vector control budget in Hidalgo County [22].

Within the LRGV region, there are over 1800 unincorporated communities known as *colonias* which are usually inhabited by families of Hispanic heritage who often live in low-quality housing and lack essential city services such as waste management, paved roads and potable water [23–25]. These systematic disparities create conditions that are favorable for *Ae. aegypti* proliferation and unfavorable for the health of community members due to deficits in multiple social determinants of health [26]. *Colonias* also have issues with social cohesiveness due in part to vacant lots [27] which contributes to them being a hard-to-reach racially minoritized group [28, 29]. These factors present barriers to engaging effectively with communities to implement vector control interventions [18, 30] in the precise environments where these interventions are most needed.

In 2016, our research team undertook a research study to test multiple mosquito control techniques in *colonias* in the LGRV. Because these interventions targeted mosquitoes in and around people’s homes, it was essential to our research design that we be able to access private properties and work directly with communities. Community engagement was thus a central component of our work. In this Research in Practice narrative, we explain our team’s experience with community engagement approaches in this context, and show how working with community leaders, following community members’ suggestions, and making subtle changes to engagement techniques improved participation in our projects and reduced dropout rate.

The inclusion of behavioral and social sciences into public health interventions cannot be overlooked, as these fields of expertise provide critical guidance when developing a project that depends on communities and public acceptance. Lessons learned here can be applied to larger efforts to work with communities to implement more effective vector control approaches, demonstrating the importance of getting early community buy-in and support.

## Main text

### Study area

Our research took place in Hidalgo County, located in the LRGV region along the US–Mexico border of South Texas, USA. Within this county, there are an estimated 800,000 people of which 90% are of Hispanic origin, 28% live below the poverty line and 19% are foreign-born individuals [31]. This region has three major points of entry from Mexico into the US (Hidalgo, Progresso and Brownsville), with over 23 million and 28 million recorded crossings for 2017 and 2018, respectively [32].

### Community selection

**Fig. 1:**
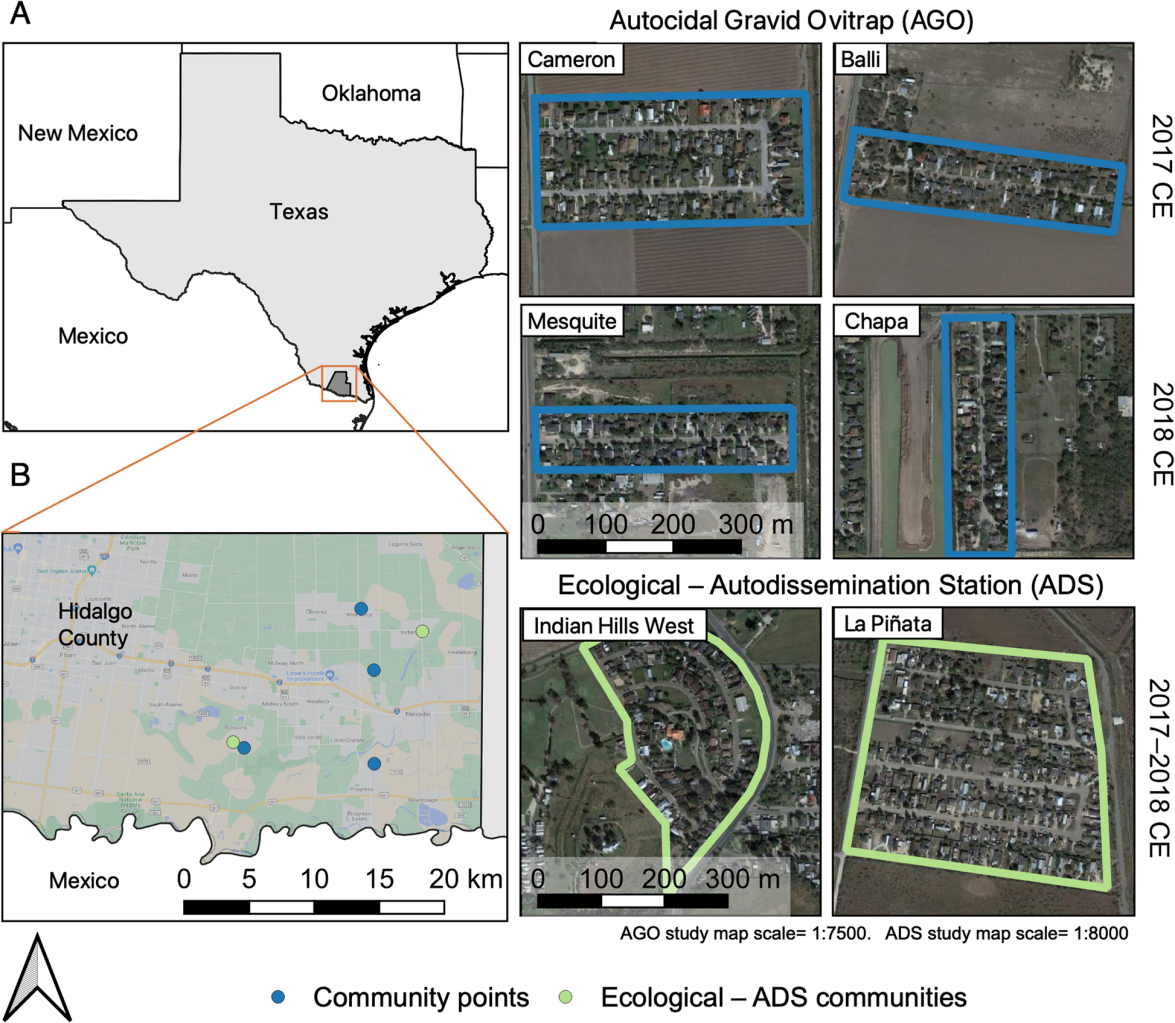
Site location of the communities involved in the Autocidal Gravid Ovitrap (AGO), Autodissemination Station (ADS) and ecological studies of *Aedes aegypti* in the Lower Rio Grande Valley, South Texas. **A** Map of Texas highlighting Hidalgo County. **B** Study communities’ location within the LRGV region, AGO study = blue dots, ecological–ADS study = green dots. **C** Communities involved in the AGO study. **D** Communities involved in the ecological and ADS studies. Community engagement (CE) refers to the year when recruitment of all houses within a community was conducted. The map was developed using QGIS 3.16 (https://qgis.org/en/site/) with Map data: Google, Maxar Technologies

Our community selection process has been detailed in prior papers [33–35]. Briefly, we used the 2010 census blocks to identify *colonias* based on a mean household income of $15,000–$29,999 that were within a 30 km radius from our field station (Texas A&M AgriLife Research and Extension Center in Weslaco, Texas) (Fig. 1). The identified *colonias* were selected based on size (e.g., range of 20 to 150 households), level of isolation from other communities or urban landscapes, and perception of safety for field personnel based on comments from local state officials. Seventeen *colonias* from Hidalgo County were initially visited for evaluation and six were selected. These *colonias* were distributed within the cities of Donna (*n* = 2), Progresso (*n* = 1) and Mercedes (*n* = 3). Our projects involved testing two developing control tools (Autocidal Gravid Ovitrap and Autodissemination Station) and two ecological projects for mosquitoes (dispersion and cryptic containers) (we expand on each one in The projects section).

### Community members

We categorized community members into two types based on their level of engagement: highly engaged persons (HEPs) and participants. HEPs received weekly household visits to check surveillance traps, participated in interviews and surveys (*n* = 23; knowledge, attitudes, and practices (KAP)), and were involved in the development of a results flyer (*n* = 8) (Table 1). Personal information was only collected from the HEP individuals involved in the KAP survey [36]. Participants included all the remaining houses within each ***c****olonia* that received the intervention and had visits on a monthly or bi-monthly visits. HEPs were randomly selected within each *colonia* with written consent provided by one of the adult household participants [34, 36]. If a HEP dropped out, we tried to recruit the neighbor to the right until a new one was recruited. HEP’s that dropped out were still invited to join the project as participants for the monthly or bimonthly visits portion of the intervention. To identify all possible houses within each community, we georeferenced all structures using Google satellite imagery (Google, Maxar Technologies) in QGIS 2.8 (https://qgis.org/en/site/) and confirmed them with field visits.

**Table 1:** *Colonias* of the LRGV with the projects carried out (Autocidal Gravid Ovitrap = AGO; Autodissemination Station = ADS), year of complete community recruitment, total active houses during the recruitment, highly engaged person (HEP) and participants

Community	Project	Year	Total active houses	HEPs	Participants
Balli	AGO	2017	40	7	18
Cameron	AGO	2017	78	6	35
Chapa	AGO	2018	27	5	19
Mesquite	AGO	2018	37	5	26
La Piñata	Dispersal	2017	151	50	–
	ADS	2018	146	15	84
Indian Hills West	Cryptic cont.	2017	79	32	–
	ADS	2018	82	10	50

### Study design and data collection

We conducted a descriptive qualitative case study on four projects carried out between September 2016 and February 2019 in the LRGV [37–39]. We employ this design to provide an account of the issues we faced with community engagement during our different projects, and try to provide causes of the problem, solutions we undertook, the outcomes of the solutions, lessons learned, and the broader theories/concepts relevant to our experience [40]. We are analyzing how gradual changes in community engagement, from minimal outreach with limited involvement from stakeholders to consultation with some input from stakeholders to researchers [41], allowed us to improve participation and retention of community members in the framework of a mosquito ecology and control research program. We kept records on the total number of occupied houses in the communities, the number of visits needed to engage community members during the house-to-house recruitment, number of dropouts for each community, and community members’ suggestions during meetings and flyer development.

### Team and expertise

A multidisciplinary team of local and international personnel, including student workers from a local University (University of Texas Rio Grande Valley), comprised the team. The expertise of our core team members varied and included local community health workers (known as *Promotoras*); local community members; members with knowledge in community engagement and expertise in Neglected Tropical Diseases; and subject matter experts in the field of mosquitoes and vector-borne diseases. Most of our core team were native Spanish speakers and fluent in English. The lived experience of our local team members provided a unique perspective into the mindset of participants living in the LRGV and the *colonias*. We leveraged this knowledge to help us understand how community members perceived outsiders and local authorities. Combining the multiple perspectives of our team we were able to develop engagement activities, educational material and surveys that were culturally appropriate and tailored for the *colonias.* This allowed us to address issues of local jargon and the inclusion of community members during the development of these tools.

### The projects

**Fig. 2:**
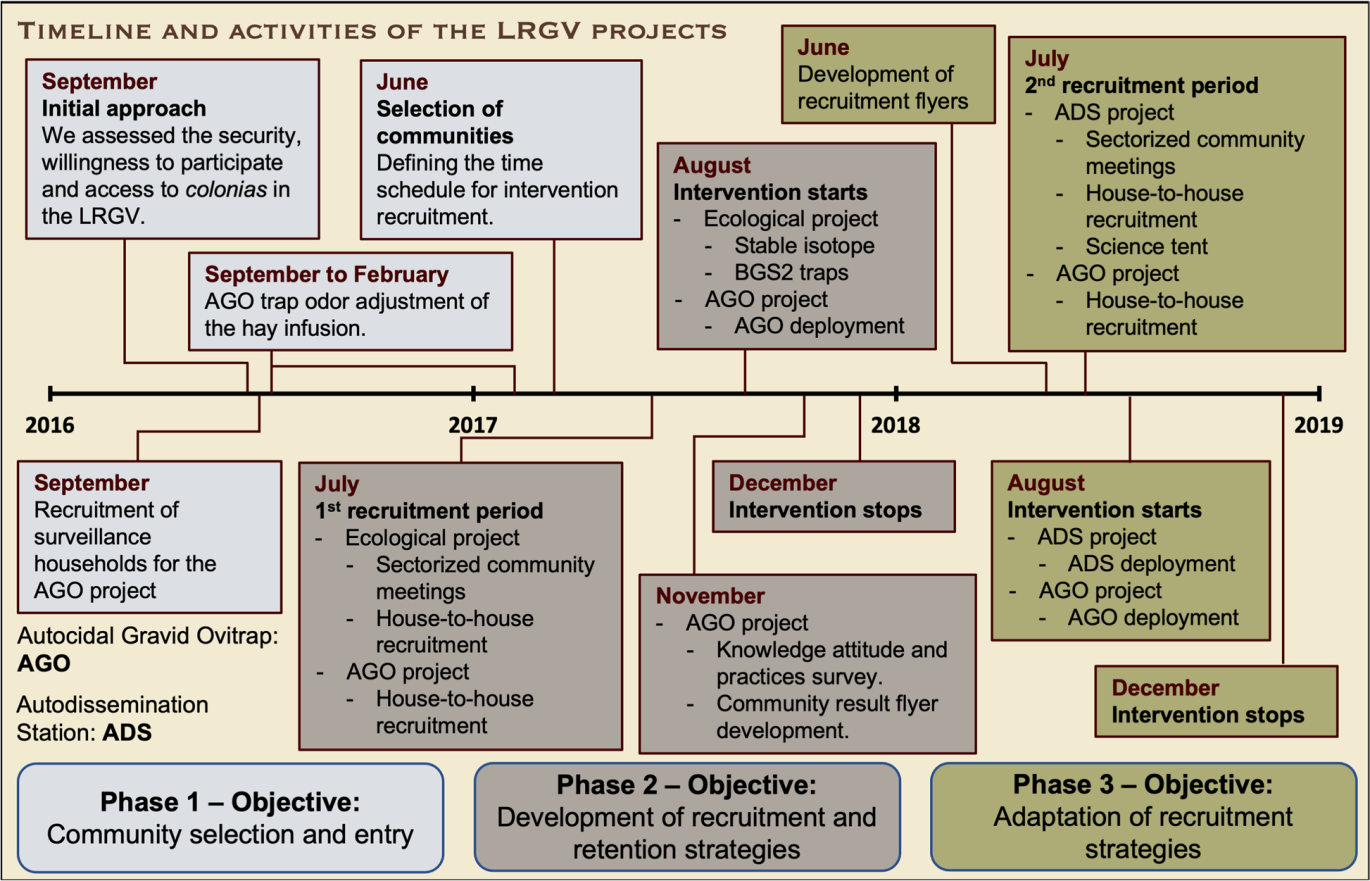
Timeline and activities carried out in the *colonias* of the Lower Rio Grande Valley (LRGV) in South Texas. Phase 1 shows the activities carried out during the initial approach of the project starting in September 2016. Phase 2 shows the 1st recruitment period starting in July 2017. Phase 3 shows the 2nd recruitment period starting in June 2018

The projects we conducted in these communities includes two intervention projects and two ecological studies focused on *Ae. aegypti*. Two control tools in development were evaluated in the intervention projects, the Autocidal Gravid Ovitrap (AGO) (Juarez et al, [30], see Supplementary Information: AGO project, for a brief description) and the Autodissemination Station (ADS), for mosquito suppression and field performance under local conditions (see Supplementary Information: Vector control traps [36]). The ecological projects involved the isotopic enrichment of container habitats to evaluate mosquito dispersal [35] and cryptic habitats. Each of these projects relied heavily on community participation, since we required permission from homeowners to enter their properties to set up traps, for either control or surveillance, and search for larval habitats. Four communities (e.g., Balli, Mesquite, Chapa and Cameron) were involved in the AGO project (Fig. 1C) and two (Indian Hills West and La Piñata) were involved in the ecological and ADS projects (Fig. 1D). In Fig. 2, we show the timeline and activities carried out for each project with three key phases (Phase 1, 2, and 3). These phases are marked by shifts/adjustments in our community engagement activities for increasing retention and participation, which we explain in our narrative in this report. Table 2 shows the processes, mechanisms and lessons learned from our community engagement activities during the different phases.

**Table 2:** Processes, mechanisms, and lessons learned for our community engagement activities during our different phases

Phases	Processes	Mechanisms	Lessons learned
Phase1	• Community selection	Recruitment of local community health workers.	Following the security recommendations of local health authorities and our local team members allowed us to generate an initial list of candidate *colonias* to evaluate. This was further delimited with the comments provided by HEPs.
		Consultation with local public health authorities.	
		Consultation with highly engaged person (HEP).	
	• Community entry	Flexible dissemination strategy	Not forcing community meetings or interaction between community members was key for recruitment of HEPs in the AGO project *colonias*.
		Field product adjustments	Adjusting the traps used to fit the requirements of participants and surveillance efforts reduced our dropout rate of HEPs.
Phase2	• Recruitment strategies	House-to-house visits	The use of flyers during the house-to-house visits served two purposes: provide information of the project and a signal for household occupancy.
		Planned meetings	*Colonias* have very different social dynamics, even for those that are geographically close.
			*Requesting personal information can negatively affect the participation rate of community members.*
	• Retention strategy	Building rapport	Schedule weekly visits for trap surveillance allowed us to have informal conversations with HEPs that ranged beyond project topics.
		Knowledge, attitude, and practices survey	The surveys allowed us to get a perspective of the gaps of information community members might have and what topics should be addressed when preparing information dissemination.
		Result flyer	Providing community members with results and allowing them be part of the development of informative flyers gave HEP’s a sense that they were doing something to help their community.
Phase 3	• Adapting recruitment strategies	Community based flyers	The use of a short recruitment flyers that were developed with the input from community members and tailored for the *colonias* involved in each project improved participation.
		Stand-in meetings	Allowing flexibility of when we could present our project and provide information in a more informal scenario, was key to reach community members that were hesitant to participate or hard to find at home.
		Science tent	The tent provided us with additional exposure in the *colonia*, and generated curiosity by some community members, but additional efforts are required to fully engage hard-to-reach persons.

#### Phase 1: community selection and entry

In this initial phase, we had a rigorous site-selection procedure for the AGO *colonias* [42] (*colonias* of the ecological projects were not part of phase 1). The long selection process in these *colonias* was because we assumed that the requirement of weekly indoor (per project objective) mosquito surveillance would affect the willingness of community members to participate. Indoor surveillance is a more intrusive process that requires that HEPs be present at the time of collection. The site-selection process involved trying to identify community leaders and having informal conversations with community members regarding the safety of their community as well as willingness to participate in a long-term mosquito project that had both indoor and outdoor surveillance. During this initial interaction we were unable to identify community leaders in these *colonias*. More interestingly, it appeared that community members had little to no contact with neighbors and some were unwilling to interact with each other.

“If I have to talk to any of my neighbors, I do not want to participate in this project” HEP.

##### A flexible dissemination strategy

From our team’s international Hispanic/Latin perspective, we did not expect to encounter this level of social isolation from community members that considered themselves Hispanics/Latins. Based on the feedback from HEPs we opted to adjust our dissemination strategy and rely on the house-to-house visits rather than group meetings for interactions and information dissemination in these *colonias*. It should be noted that Latin immigrants experience social isolation in the US that might prevent them from stablishing social support networks [43], and harder immigration policies appear to exacerbate this effect in cities with high migrant populations [44]. This is something we believe might be happening here. Even though community members did not acknowledge it, we perceived some of them were worried we might be working for a law enforcement agency in particular the Border Patrol. For example, some of them asked “jokingly” if we had microphones inside our mosquito traps, we responded by showing them the trap and filling it with water. To prevent any confusion, all team members and visiting scholars always wore university associated clothing and avoided green colored attire to decrease the perception that the team was associated with the Border Patrol (who wear green uniforms) or blue colored apparel to decrease the perception of affiliation with Immigration and Customs Enforcement.

##### Field product adjustment

Our recruitment procedures were successful in helping us avoid communities that might pose a security risk for our field team (i.e., presence of drugs, gangs, angry dogs and/or comments from community members regarding safety) and the main issue we encountered was that several HEPs objected to the odor produced by the AGO trap both indoor and outdoor. This represented a major issue since the trap needs to be standardized when used in different regions to allow comparisons across sites, and changes to it need to be done carefully. Adjustments for the formulation that produced the odor were made with the help of HEPs to identify a dose that did not disturb residents but still attracted female mosquitoes. This type of collaboration when designing and testing novel ovitraps has been proven useful in other contexts as well [45]. The help from HEPs allowed us to generate a formulation that was improved for the Texas heat, which was used for the reminder of the project. This formulation has also been used by another group in Texas that showed no effect on trapping rate between the original dose and this modification [46]. At the end of this phase we had a 44% (16/36) dropout rate [34]. The main reasons for dropping out of the study during this phase were the odor of the trap (8/16) and the requirement for the indoor surveillance (5/16).

#### Phase 2: development of recruitment and retention strategies

##### House-to-house visits

**Fig. 3:**
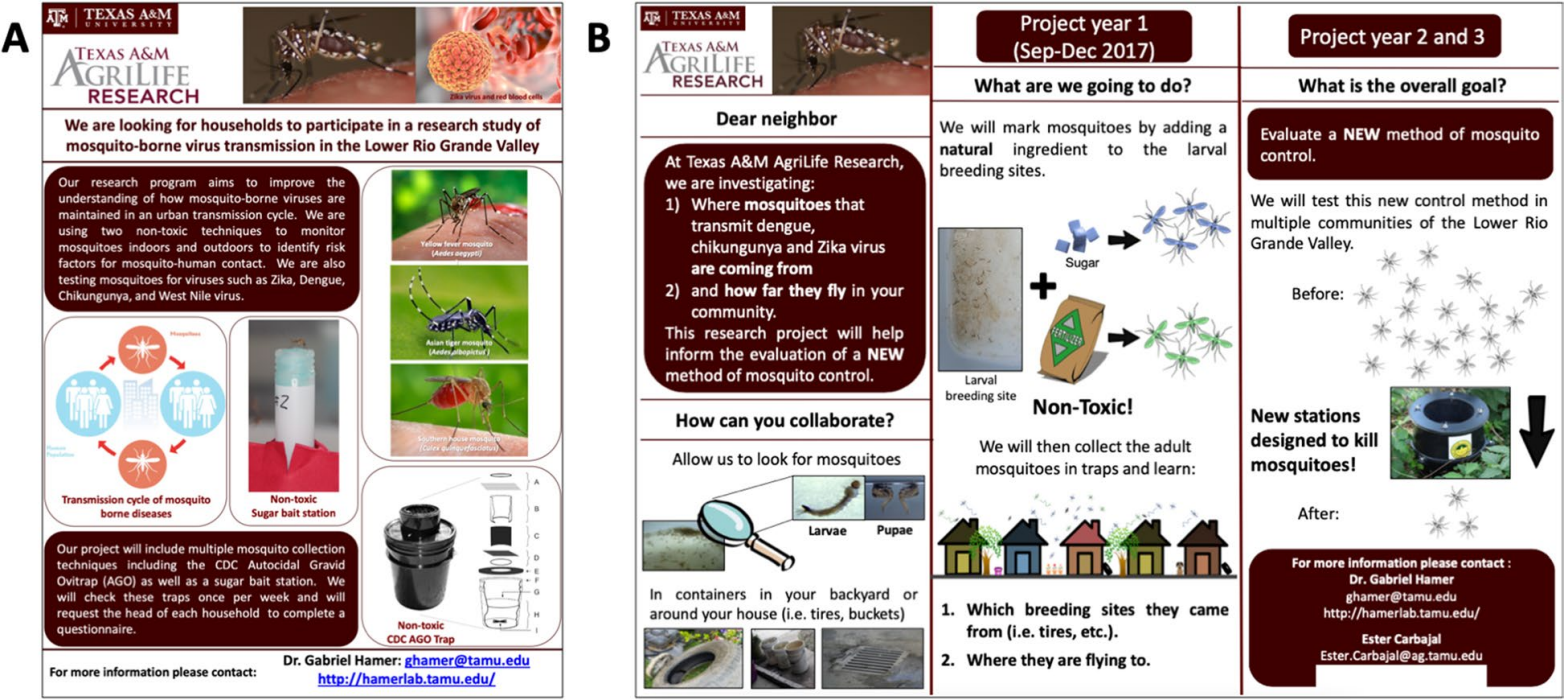
Information flyer used during phase 2. **A** Information flyer Autocidal Gravid Ovitrap project. **B** Information flyer ecological projects. Flyers generated using PowerPoint (Microsoft, USA)

Moving into the second phase of the project, a house-to-house approach for all recruitment activities based on the comments from HEPs of the AGO *colonias* regarding community meetings was chosen. We determined that each household would be visited at least three times (e.g., morning: 9:00–11:00 am; afternoon: 1:00–5:00 pm; and weekend: 10:00 am to 2:00 pm), giving every household an equal opportunity to join the project. During recruitment we conducted trap demonstrations on-site and reviewed the different procedures carefully in either English or Spanish, as requested. In this phase, we also used informative flyers (Fig. 3A-B) that were developed by the team members, with no community input. Flyers served for two purposes: 1) provided an overview of the project with our contact information to community members and 2) served as a signal to our recruitment team if a house was unoccupied. If no one was found in the house during the first visit, we left the flyer hanging on a visible area of the door (e.g., above the doorknob) or gate (e.g., in the lock); if this signal remained in the same place after the third visit, we considered the house empty. On several occasions, the flyer was picked up, and voices were heard inside of the household, but no one responded directly to our knocks.

The *colonias* in the ecological projects were approached in July 2017 (see Supplementary Information: ecological projects, for a brief description), 1 month before field activities. We planned to recruit as many households as possible and randomly select the HEPs. During initial visits with community members, we assumed (as observed in the AGO *colonias*) the lack of social integration. However, in contrast to the *colonias* included in Phase 1, we found that there was more social interaction and integration in these Phase 2 communities, with several groups in each *colonia* organized around family association or shared common interest (i.e., mothers from school children, social friends, etc.). Members within these groups would have regular communication with each other by either WhatsApp groups, a mobile communication application, or visits to each other’s house.

Our first encounter with this fact was when a lady approached us because we had left a flyer in the house of another member of their WhatsApp group chat (mothers of children of La Piñata that went to the same school), and she wanted to clarify if our project would be producing stronger mosquitoes.

“You guys left this flyer in the house of one of my friends and we are worried you are going to be adding fertilizer to the water of tires and producing stronger mosquitoes” HEP.

This interaction allowed us to program the first community meeting in the *colonia*, which we used to alleviate the concern regarding the production of stronger mosquitoes in their community and fully address all the details of our projects.

##### Planned meetings

This encounter showed us that we needed to search for other groups within these *colonias* that serve as community gatekeepers or leaders that might help us disseminate information [47]. We were able to identify two groups and three group leaders, and we conducted pre-meetings with gatekeepers and leaders to schedule presentations. Our first meeting with a group had a low attendance (*n* = 4) in relation to the expected participants that would arrive based on the WhatsApp group (approximately 20). During another meeting we had with a group leader we perceived a lot of distrust from the leader towards our intentions for surveying mosquitoes, nonetheless she agreed to our project and mentioned another community gatekeeper for a sector of the *colonia* that we should also present the project to. This gatekeeper turned out to be essential for communicating with other community members regarding why our traps should not be damaged or stolen.

“There are some kids in the *colonia* that do mischiefs but if they understand the need of the traps nothing will happen to them” HEP

During our different meetings, we presented the projects, discussed study limitations, and alleviated doubts from community members. This process showed us two key aspects of these *colonias*. Firstly, some of these groups did not interact with each other, in one case one group argued against the inclusion of another group in the project. We explained the need of having as many members as possible of the community. Secondly, some of these groups had a leader whose approval was necessary to effectively recruit households.

“Did the house in the corner agree? If so, you can place the traps” Participant

This suggested that approval from the leader was necessary. However, we still relied heavily on house-to-house recruitment since these groups were only a small portion of the people living in the whole *colonias*.

**Fig. 4:**
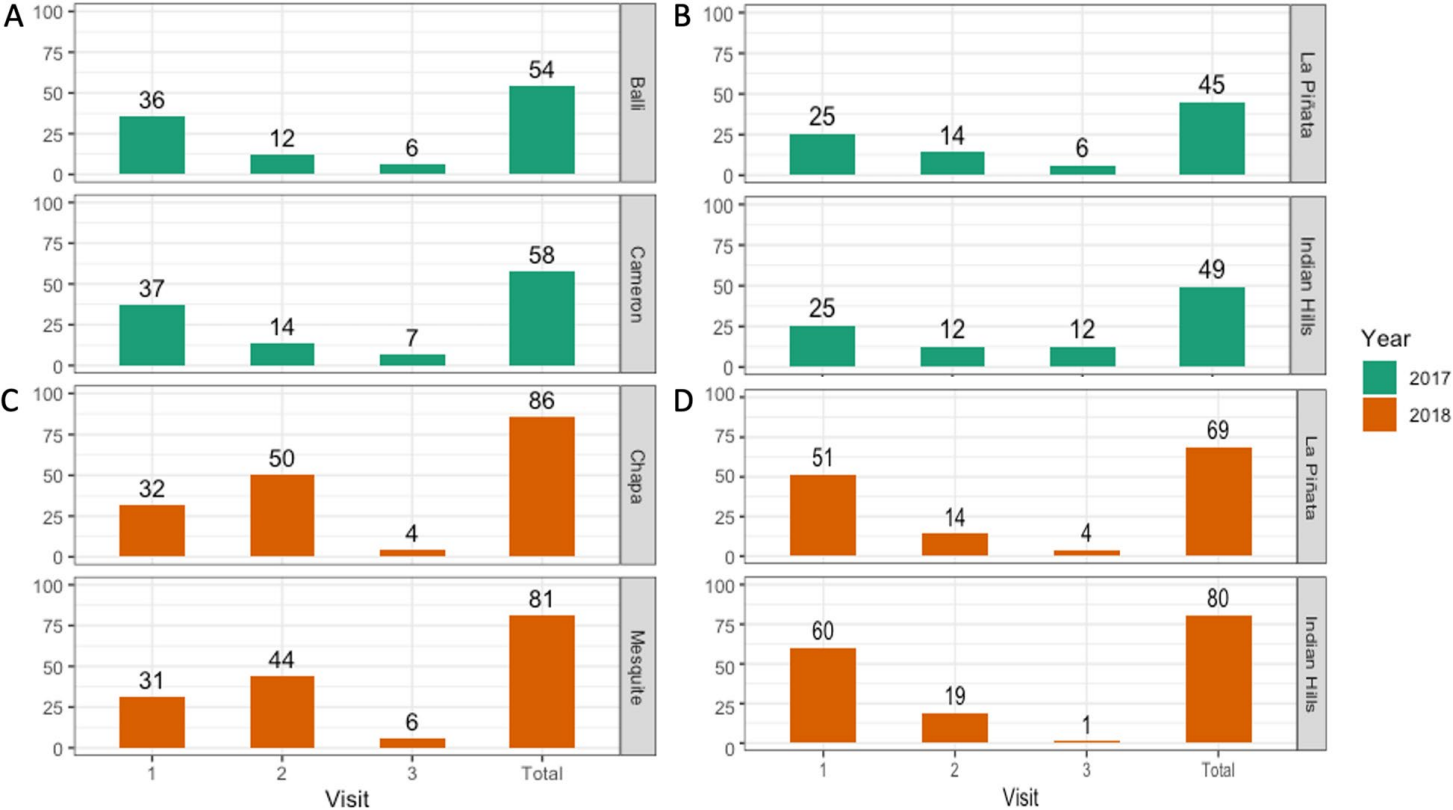
Recruitment rates for the Autocidal Gravid Ovitrap and ecological/Autodissemination Station projects in *colonias* of the Lower Rio Grande Valley. Our team visited every household in each *colonia* up to three times to recruit them for each project. Results are presented as the percent of the households in each *colonia* that agreed to participate after the first, second, and third visits, and in total. **A** Recruitment results for the AGO project, 2017. **B** Recruitment results for the ecological projects, 2017. **C** Recruitment results for the AGO project, 2018. **D** Recruitment results for the ecological projects, 2018

For the AGO project, after the third house-to-house visit, we recruited between 48 and 55% of the available households from the target *colonias* (Fig. 4A). Informal discussions with some community members that were recruited as participants showed that some of them did not open the door during the first visit because they thought we were either 1) selling something, 2) debt collectors, or 3) members of a religious organization. The recruitment results for the ecological projects were similar to those observed for the AGO project, with 42% (La Piñata) and 49% (Indian Hills West) of community members recruited (Fig. 4B). Overall, the results showed us that the recruitment method for Phase 2 was not sufficient to effectively reach most of the community members in all the *colonias*. We were targeting an 80% community participation based on other studies that had used AGO’s as a control tool [48], which would allow us to compare results between regions.

##### Building rapport with HEPs for long-term enrollment (AGO)

Since our presence in these *colonias* was going to be yearlong (apart from the last 2 weeks of December and the first week of January, due to the Christmas/New Year’s holiday) our goal was to maximize retention of HEPs. As part of our strategy for retaining households, we had informal conversations with HEPs during our weekly visits, which could last between 15 (mainly operational) to 45 minutes. Topics ranged from the perception of their community, our study, local vector control activities, and personal issues they encountered during the week. We also provided information about seasonal mosquito abundance in their home, community, and region. This information was given whenever the homeowner requested it, as well as to all households at the start of 2018 and after the project ended in 2019.

To better understand the perception of HEPs regarding mosquitoes, including their diseases and control measures, and our project, we carried out a Knowledge, Attitude and Practices (KAP) survey in November 2017 and 2018 [36]. Other studies have shown that the use of formative research has helped improve information sharing, promote understanding and increase participation [40, 49]. The results obtained from the 2017 KAP showed that community members considered the use of television and flyers as the best methods for communication in the *colonias*. The KAP also allowed us to generate an initial draft of an informative result flyer for Phase 2 to work with community members (see Supporting Fig. S1A – B). The final version showcased information that community members perceived as critical, such as increasing the size of the images used for the seasonality, emphasizing the Centers for Disease Control and Prevention (CDC) webpage, and showing the actual size of the mosquitoes we were studying. They also suggested decreasing the amount of text and increasing the size of the greeting message. This flyer was distributed in February 2018 to all community members in the different projects regardless of involvement in the studies. Overall, community members appeared more receptive to this flyer, even those that had not participated in the project were interested in having us explain the flyer and our activities. We even had some HEPs that requested more than one flyer so they could show it to other people. Finally, we provided HEPs with a $5 gift card from a local supermarket on four occasions, two times in both 2017 and 2018. These were provided in August and December as a token of appreciation to homeowners for their consistent support, taking into consideration the minimum wage per hour of the region. This type of compensation must be done carefully, since it could lead to bullying and discrimination by other participants [50]. At the end of phase two (May 2018) we had a 4.45% (2/44) dropout rate [30], a 90% decrease in dropout compared to Phase 1. We did not have additional dropouts after this time point.

#### Phase 3: adapting a recruitment strategy

##### Adjusting community engagement tools

**Fig. 5:**
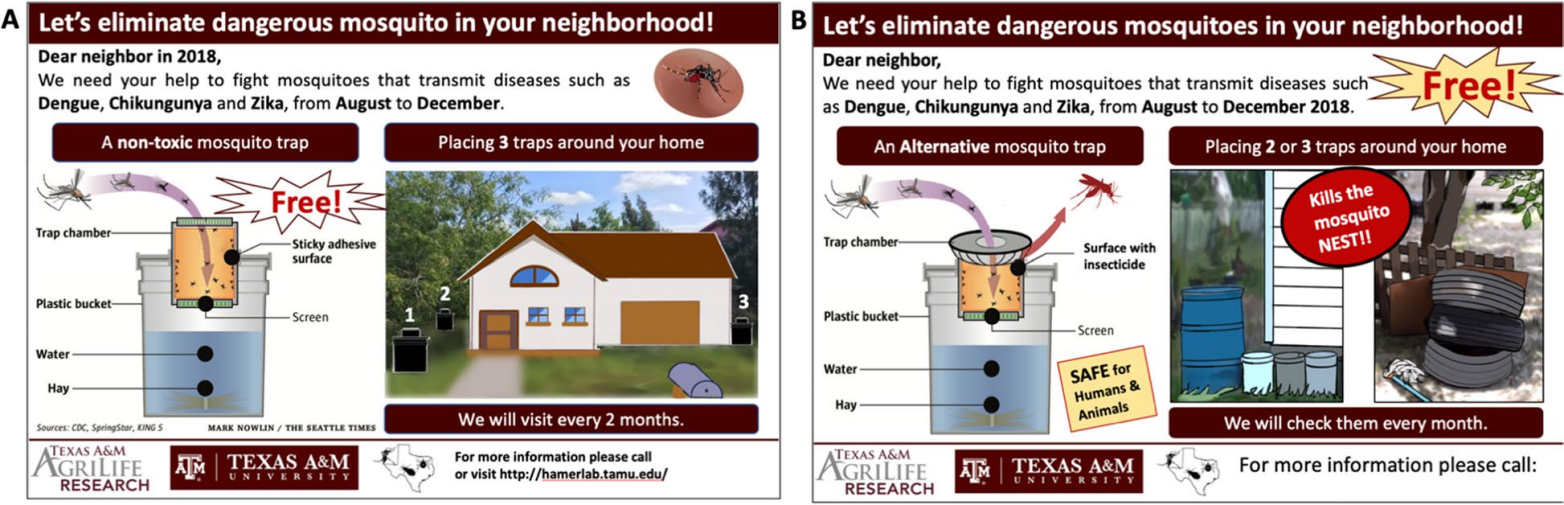
Information flyer used during the recruitment of Phase 3. **A** Flyer for the Autocidal Gravid Ovitrap project. **B** Flyer for the Autodissemination Station project. Flyers were generated using PowerPoint (Microsoft, USA)

In both projects we decided to slightly modify the recruitment process by conducting the third visit after 5:00 pm, since we noticed participants worked and several were unavailable before this time. We also developed a brief recruitment flyers (Fig. 5A-B) based on feedback from participants to improve aesthetics, clear objectives of the project, and clarity of words. The first recommendation from community members was to make it clear that the trap was free and to clarify the procedures involved, such as a reset visit every 1 or 2 months by the study team. Another key comment was to clarify the safety of the trap. In Fig. 5B we show the phrase “Kills the mosquito nest”, which was an idea that came from one participant when we explained the project during a meeting.

“So, this trap works as the cockroach trap that kills the nest” Participant

##### Stand-in meetings

The ADS project had the added component of community meetings. In contrast to the AGO study, both HEPs and participants were recruited simultaneously. We modified our approach to meetings based on a comment from a community gatekeeper that carrying meetings would be easier if we approached them if they were already “hanging out” which usually happened after 5 pm. This recommendation proved effective since we were able to successfully present our project to two groups that were already gathered. In one meeting the group leader acknowledged our presence in the *colonia* the previous year commenting that the trap we used for surveillance reduced the number of mosquitoes he had outdoors. This same group leader was also able to get other neighbors on board with the project that were not present during the meeting, even those that were not living in the *colonia* at the time, but he had access to their property. We tried to ensure the purpose of the study was well understood by all attendees (see Supplementary Information: ADS project, for a brief description).

The meetings in La Piñata allowed us to determine the need for extra engagement activities in the community to stablish trust and adequately disseminate information within the *colonia* [47]. During the meetings we perceived that some community members were still worried we could work for law enforcement. On one occasion, that we know of, when we left the community for our base of operations, a vehicle followed us and watched us from a distance as we unloaded the mosquito traps. Suspicion towards project personnel has been observed in other low-income settings [51] which may ultimately compromise the overall project [52]. To reduce the level of suspicion from community members we discussed with community gatekeepers the option of a science tent at the entrance of the community to gain exposure in the *colonia*. They were receptive to the activity, so this was planned for the Saturday before the house-to-house visits started. The tent had information regarding our study, the ADS trap, live mosquito larvae and activities for children. Nonetheless, this added component was not sufficient to achieve the desired coverage of the ADS intervention. This revealed that our strategy needs to be further improved to achieve trust with community members. One approach could be to tailor the results to community members or follow-ups with one-on-one meetings to identify changes [47].

In both the AGO and ADS projects recruitment rates increased in this phase. The AGO project had recruitment rates between 81 and 86% of households. Comparing recruitment for Phase 2 and Phase 3, we had similar results for the first visit (average of 37 and 32% respectively) but saw a large increase in recruitment at the second visit with an average of 47% in 2018 (Fig. 4C) compared to an average of 13% in 2017. In the ADS project we recruited between 69 and 80% of community members. When comparing the recruitment Phase 2 and Phase 3 we observed an increased average of 30% on the first visit for 2018 (Fig. 4D). The success in recruitment in Indian Hills West could be due to our presence being more widely acknowledged by community members. Since participants in this *colonia* would talk to us more often than in La Piñata. Interestingly, for both projects in this recruitment phase, we had to re-enroll between 2% (AGO) and 5% (ADS) of households. When we arrived to service the traps, different participants were living in the house. In some cases, participants wondered what the traps were for and in others, they had already been told about our project. We believe some of these households host transient populations that might only spend a short time in the *colonias*. Re-enrollment usually happened after 2 months of the traps being deployed.

### Strengths and limitations

The engagement of stakeholders and community members in public health interventions is advocated as a key component of improving the health and well-being of disadvantaged and marginalized groups [39]. For novel methods of vector control, early involvement of key participants may allow us to assess acceptability of new approaches and to detect problems that may lead to public rejection of certain technologies [53, 54]. More importantly, engaging with affected populations allows us to gain in-depth knowledge of the ecological, biological, political, and social complexities in which the novel vector control approach would be implemented. Community engagement does not have a one size fits all framework that all projects could follow [55], even in situations like ours where communities are geographically close. However, there are several principles or activities that projects might undertake to improve their community engagement procedures. For instance, the inclusion of local human resources proved a great advantage when selecting communities and understanding the local jargon to provide clarity when communicating with participants, something that has also been observed for malaria elimination [49]. Adjusting our dissemination strategy based on the various social structures found in the *colonias* helped us build trust and reach more community members, this type of flexibility has also proven useful in designing successful HIV community engagement projects [47]. The KAP’s survey allowed us to gain in-depth knowledge of the perspective the community members had regarding our project and mosquitoes, ultimately helping us improve the information presented in our flyers, the use of KAP’s has also been used to guide community based interventions for Chagas disease [56].

Community engagement activities need to be culturally appropriate and sensitive to effectively reach community members from underserved populations [57] which might be affected by different disparities and barriers (e.g., budgetary constraints, social cohesiveness). From the different projects we conducted related to mosquito ecology and control, we show that even with a limited budget for community engagement, the inclusion of such activities allowed us to improve community participation and retention and engage transient community members in the *colonias* of the LRGV along the US-Mexico border in Texas.

Undertaking community engagement activities can feel overwhelming especially for local vector control programs that may lack trained personnel in the social and behavioral sciences. Understanding what community engagement is might be confusing since there is a variety of definitions and models available for program implementation [53, 55, 57]. In the case of the *colonias,* external investigators might think that these community members would share the same perspectives or belief systems since most are of Hispanic heritage. However, the reality is that community members migrated from a range of different countries, and have different immigration statuses, ethnicities, languages, or dialects (e.g., North Mexican Spanish, Guatemalan Spanish, TexMex, etc.), and cultural nuances. This shows a clear need to understand the needs of our communities at a local level while taking into consideration that mistrust from marginalized communities is common [58] and was something that we were able to observe throughout our studies within the *colonias*. Community engagement is a long process that cannot be rushed, it requires time and effort from team members to build trust. In our case showing up each year proved effective, since community members had more time to assess our safety towards their community and understand the benefits our projects could bring.

In our study, we continuously worked with community members to develop culturally sensitive recruitment materials and to better understand the social relationships and power dynamics. Some limitations of our projects were that we were unable to fully involve community members in the design and type of intervention to be used. We had budgetary constraints for the inclusion of a larger sample of community members along the LRGV, as well as for the materials we could develop and use. Finally, our work has socio-geographic barriers that might not exist elsewhere so we cannot generalize our results to all *colonias* along the US-Mexico border.

We observed that the inclusion of community members for the outreach material and the type of communication to use (flyers) proved very useful when evaluating the efficiency of the AGO intervention, which showed that this trap is dependent on density in an area that ultimately depended on community participation [30]. The inclusion of community engagement activities in our multiple projects increased stakeholder engagement and acceptability and allowed us to conduct robust science in communities that might be considered as hard to reach. This allowed us to also elucidate novel ecological features of mosquitos such as a longer range of dispersal for the region [35] and risk factors associated with female mosquito abundance [36]. These studies would not have been possible to conduct without the support and willingness of community members to allow us to enter their homes either on a weekly, monthly, or bimonthly basis. Trust from participants was crucial since on many occasions we were authorized to go into their properties without them being present, providing us with consistency in our surveillance data.

## Conclusion

With community engagement increasingly viewed as a critical piece of any research project for promoting recruitment and retention [59], there is a need to understand its challenges when applied in different sociodemographic settings. Of course, community engagement must be done thoughtfully and carefully – helpful guidelines for engaging with communities are provided in [55] for example. The results drawn from our projects show that community engagement activities should be a key component of any vector-borne disease research project, effective local vector control program, or other public health intervention for building trust, respecting community views and gaining permission. Community engagement should be a standard ethical good practice strategy from the start of any project [60]. Among vector control programs with budgetary constraints, our studies show that small efforts in community engagement can have a positive impact on their mitigation efforts.

## Supplementary Information

**Additional file 1.** Supplementary Dataset, raw information of recruitment.

**Additional file 2.** Supplementary Information, additional description of methods section for project description.

## Data Availability

All data generated or analyzed during this study are included in this published article [and its supplementary information files].

## Abbreviations

AGO: Autocidal Gravid Ovitrap
ADS: Autodissemination Station
LRGV: Lower Rio Grande Valley
KAP: Knowledge Attitude and Practice
HEP: Highly Engaged Person
