# Supplementary material for: The unreachable doorbells of South Texas: community engagement in colonias on the US-Mexico border for mosquito control
Source: BMC Public Health. 2022 Jun 13;22:1176. doi: 10.1186/s12889-022-13426-z (PMC9190097; doi:10.1186/s12889-022-13426-z)
Supplement: Supplementary file 2 — Additional file 2. Supplementary Information, additional description of methods section for project description. [file 12889_2022_13426_MOESM2_ESM.docx]

**Supplementary information**

**Vector control traps**

These control tools rely on exploiting the oviposition behavior of female mosquitoes by simulating artificial larval habitats. Both units consist of a black container that holds water and uses hay as an odor attractant. The top of the unit either contains an adhesive glue to trap and kill mosquitoes (AGO) or a larvicide to be picked-up and disseminated by female mosquitoes to another container larval habitat (ADS).

**AGO project**

Briefly, our initial approach started in September 2016 through June 2017. In this time point we upscaled our weekly surveillance efforts to an average of five engages (with one indoor and outdoor trap) and sustained this until December 2018. The intervention took place between August through December for both 2017 and 2018, with only two *colonias* having the intervention per year. The project required that participants had three AGO traps deployed in the peridomicile of the house with bi-monthly maintenance of the traps.

**Ecological projects**

We conducted two main projects related to the ecology of *Ae. aegypti* mosquitoes in the *colonias* of La Piñata (natural dispersal from container habitats [29]) and Indian Hills West (cryptic containers). These projects consisted of enriching discarded container habitats with stable isotopes for tracking adult mosquitoes throughout the communities. Both projects were conducted from August to December 2017, with weekly mosquito surveillance using BG sentinel 2 (BGS2) traps (BioGent, Germany) in the engages.


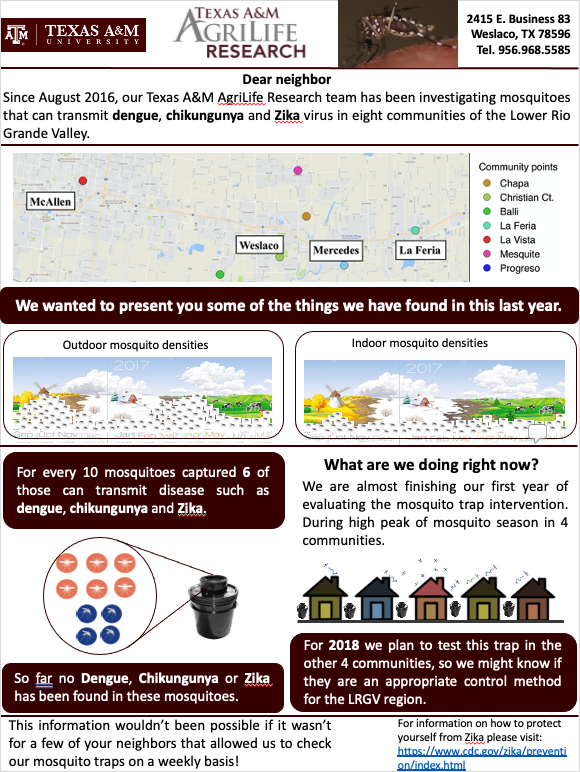

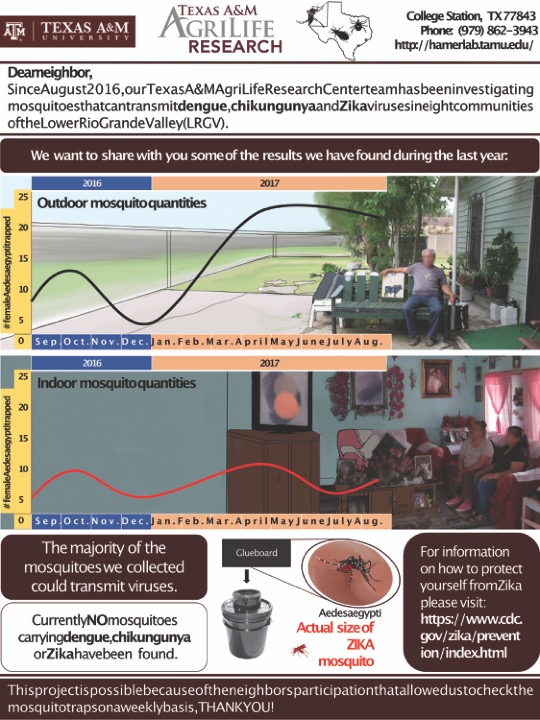


Figure S1. Autocidal Gravid Ovitrap (AGO) result flyer. A) First draft version of the result flyer with no input from community members. B) Result flyer developed by community members and research team with information regarding the study, mosquitoes, and their diseases. All flyers had a Spanish version in the back. Flyer generated using PowerPoint (Microsoft, USA).

**Autodissemination station (ADS) project**

The ADS project consisted of an intervention testing a novel mosquito trap that relied on the insect growth regulator pyriproxyfen for mosquito suppression. The project was conducted in the communities of La Piñata and Indian Hills West, but in 2018. As with the AGO, the procedure involved with this intervention required for us to deploy an average of 3 ADS units per participant in the peridomicile. We carried out weekly surveillance of mosquitoes with the use of BGS2 traps in the peridomicile of engages. The intervention activities were carried out from August to December 2018.
